# Caspase-11 regulates the tumour suppressor function of STAT1 in a murine model of colitis-associated carcinogenesis

**DOI:** 10.1038/s41388-018-0613-5

**Published:** 2018-12-11

**Authors:** Brian Flood, Joan Manils, Ciara Nulty, Ewelina Flis, Sinead Kenealy, Gillian Barber, Joanna Fay, Kingston H. G. Mills, Elaine W. Kay, Emma M. Creagh

**Affiliations:** 1 Trinity Biomedical Sciences Institute, School of Biochemistry & Immunology, Trinity College Dublin, Dublin 2, Ireland; 2 Royal College of Surgeons in Ireland and Beaumont Hospital, Dublin 9, Ireland

**Keywords:** Inflammation, Colorectal cancer

## Abstract

Murine inflammatory caspase-11 has an important role in intestinal epithelial inflammation and barrier function. Activation of the non-canonical inflammasome, mediated by caspase-11, serves as a regulatory pathway for the production of the pro-inflammatory cytokines IL-1β and IL-18, and has a key role in pyroptotic cell death. We have previously demonstrated a protective role for caspase-11 during dextran sulphate sodium (DSS)-induced colitis, however the importance of caspase-11 during colorectal tumour development remains unclear. Here, we show that *Casp11*^−/−^ mice are highly susceptible to the azoxymethane (AOM)-DSS model of colitis-associated cancer (CAC), compared to their wild type (WT) littermates. We show that deficient IL-18 production occurs at initial inflammation stages of disease, and that IL-1β production is more significantly impaired in *Casp11*^−/−^ colons during established CAC. We identify defective STAT1 activation in *Casp11*^−/−^ colons during disease progression, and show that IL-1β signalling induces caspase-11 expression and STAT1 activation in primary murine macrophages and intestinal epithelial cells. These findings uncover an anti-tumour role for the caspase-11 and the non-canonical inflammasome during CAC, and suggest a critical role for caspase-11, linking IL-1β and STAT1 signalling pathways.

## Introduction

Colorectal cancer (CRC) represents the second most common incident solid organ cancer in both females and males, with an estimated annual incidence in 2008 of 1.2 million worldwide [1]. Innate immunity significantly contributes to acute inflammation, and has a critical role in the pathogenesis of inflammatory bowel diseases (IBDs) [2], which are risk factors for the development of colitis-associated cancer (CAC) [3]. Although not fully understood, the progression of IBD to CAC is associated with persistent immune cell infiltration and inflammatory cytokine production, under the regulation of inflammation-associated transcription factors, such as NFκB and STAT1 [4].

Canonical inflammasome-mediated caspase-1 activation results in the cleavage and secretion of the pro-inflammatory cytokines IL-1β and IL-18 [5, 6], and is also associated with pyroptosis, the pro-inflammatory form of cell death [7]. The non-canonical inflammasome, mediated by caspase-11, serves as an additional regulatory pathway for caspase-1 maturation of IL-1β and IL-18 in response to Gram-negative bacteria, such as *Escherichia coli* and *Citrobacter rodentium*, which introduce LPS into the host cytoplasm during infection [8, 9]. Pyroptosis is also inducible by caspase-11, independently of canonical inflammasome assembly, in response to cytosolic LPS [10, 11].

Caspase-11 is unique among caspases in that it is highly regulated at levels of transcription upon the activation of inflammatory responses. Pro-caspase-11 is inducible upon stimulation with activators of innate immunity, including LPS, IFNγ, and type I IFN [12–15]. In the context of intestinal inflammation, we and others have demonstrated a protective role for caspase-11 and the non-canonical inflammasome in controlling mucosal integrity during DSS-induced colitis [12, 16, 17]. The DSS-colitis phenotype of *Casp11*^−/−^ mice is very similar to those of mice deficient in canonical inflammasome components, caspase-1, ASC and NLRP3 [4, 18]. In humans, intestinal expression of caspases-4 and -5, orthologs of murine caspase-11, is significantly enhanced in colon tissue from IBD and CRC patients [19]. Of note, epithelial expression of caspases-4 and -5 is exclusive to neoplastic colon tissue, and have potential as CRC biomarkers [19]. Thus, it is tempting to speculate that inflammatory caspases-4 and -5 have a functional role during CRC.

Canonical inflammasome components, including caspase-1, ASC, NLRP3, NLRC4, NLRP1 and AIM2 have been shown to significantly attenuate inflammation and tumorigenesis in the murine CAC model [4, 18, 20–27]. With the exception of AIM2, the protective effects of inflammasomes during CAC occurs by regulating IL-18 production [4, 18, 20–25]. IL-18 secretion stimulates intestinal epithelial cell (IEC) regeneration and repair, and improves colon barrier function, which may explain the enhanced susceptibility of inflammasome-deficient mice to CAC [28]. Given the essential role for caspase-11 and the non-canonical inflammasome in the regulation of IL-18 production during DSS-colitis, we hypothesised that caspase-11 may also be essential for the production of intestinal IL-18 and IL-1β during CAC. To explore this hypothesis, we investigated the role of caspase-11 in the pathogenesis of CAC using the AOM-DSS model in *Casp11*^−/−^ mice.

This study demonstrates that caspase-11-deficient mice have an increased susceptibility to colitis-associated tumourigenesis. We show reduced intestinal IL-1β production and impaired STAT1 activation in AOM-DSS-treated *Casp11*^−/−^ mice, consistent with decreased IEC death and increased IEC proliferation, when compared with similarly treated WT mice. We also demonstrate a requirement for caspase-11 during IL-1β-mediated STAT1 activation in IECs. Collectively, our data demonstrate that caspase-11, and a functional non-canonical inflammasome, is required for the tumour suppressive role of STAT1 during experimental CAC.

## Results

### Increased susceptibility of Casp11^−/−^ mice to experimental CAC

**Fig. 1:**
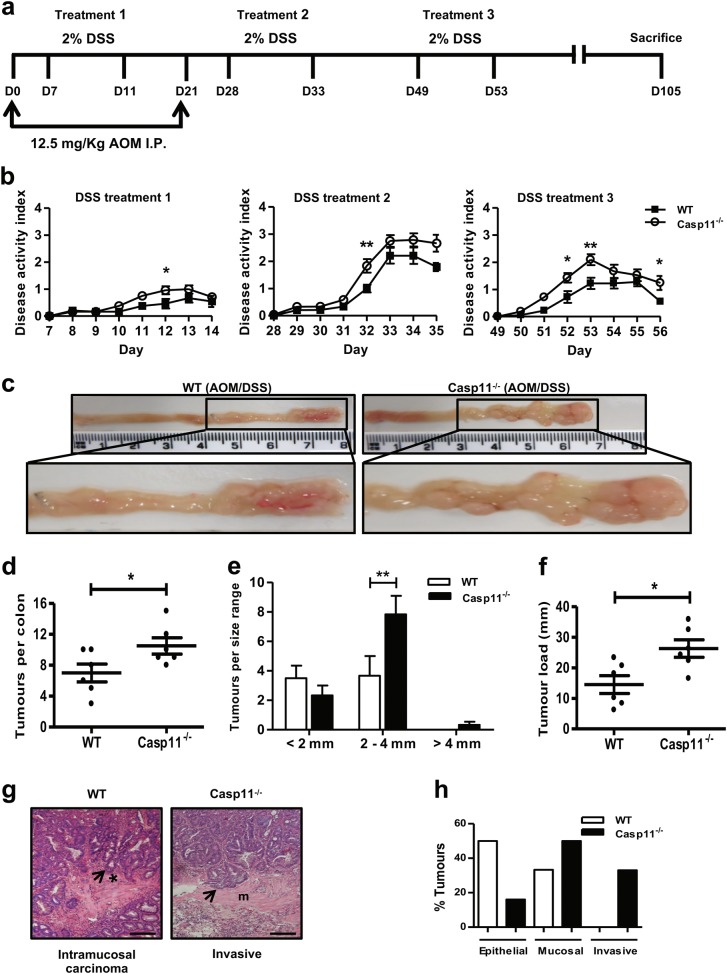
Increased susceptibility of Casp11-/- mice to experimental CAC. **a** Schematic representation of the AOM/DSS administration regime to induce tumour progression. WT and *Casp11*^−/−^ mice were injected intraperitoneally (IP) with 12.5 mg/kg AOM on days 0 and 21 followed by 3 cycles of 2% DSS (w/v). Mice were culled on day 105. **b** Disease activity index (combination of weight loss, stool consistency and intestinal bleeding scores) during each DSS administration. Data represent mean ± SEM of *n* = 6 AOM/DSS-treated mice for both groups; *p *<* 0.05; **p *<* 0.01 (two-way ANOVA followed by the Bonferroni post test). **c** Representative macroscopic view of the AOM/DSS-derived tumours in AOM/DSS-treated WT and *Casp11*^−/−^ mice, enhanced image magnification facilitates the visualisation of defined adenomas. **d**–**f** Total tumour numbers were macroscopically enumerated in AOM/DSS-treated WT and *Casp11*^−/−^ whole colons (**d**); measured and assigned a size range (**e**); and each mouse was subsequently assigned a tumour load (calculated by the addition of each tumour diameter) (**f**). Data represent mean ± SEM of *n* = 6 AOM/DSS-treated mice for both groups; *p *<* 0.05; **p *<* 0.01 (two-tailed independent Student *t-*test). **g** Representative images of WT and *Casp11*^−/−^ AOM/DSS-treated H&E stained colon tissue displaying intramucosal carcinoma and invasive carcinoma (scale bar = 200 μm). **h** Tumours from AOM/DSS-treated mice displaying epithelial, mucosal and invasive carcinomas, as a percentage of total tumours. H&E staining; *muscularis mucosae; m, submucosa

To determine whether the increased intestinal inflammation in *Casp11*^−/−^ mice led to increased colitis-associated colorectal tumourigenesis, *Casp11*^−/−^ mice and their wild-type (WT) littermates were treated with the DNA methylating agent AOM (12.5 mg/kg) and three cycles of 2% DSS interspaced with normal drinking water (Fig. 1a). Mice were monitored for clinical signs of gastrointestinal disease (weight loss, diarrhoea and rectal bleeding, Supplementary Figure 1a). *Casp11*^−/−^ mice consistently scored higher than their WT counterparts for diarrhoea and rectal bleeding, which contributed to overall higher disease activity scores across each of the DSS treatments (Fig. 1b, Supplementary Figure 1b). At the experimental endpoint, 15 weeks after the initial AOM injection, macroscopic evaluation of dissected and washed colons indicated the development of well-defined tumours in WT and *Casp11*^−/−^-treated mice (Fig. 1c). The majority of tumours were located in the distal colon in both genotypes, although *Casp11*^−/−^ mice also displayed numerous tumours in the mid-colon region (Fig. 1c). Further evaluation of tumour number and size revealed that tumours in *Casp11*^−/−^ mice were larger and of higher incidence than those observed in WT mice, with a significantly greater tumour load in *Casp11*^−/−^ mice (Fig. 1d-f). Histopathological examination revealed that tumours from *Casp11*^−/−^ mice were more advanced than WT tumours. 33% of *Casp11*^−/−^ mice displayed aggressive, invasive carcinomas (as indicated by submucosal invasion), which was not observed in WT mice (Fig. 1g, h). Furthermore, tumour tissue from 50% of *Casp11*^−/−^ mice displayed extension of severely dysplastic regions below the muscularis mucosae, compared with 33% of WT mice (Fig. 1g, h). These observations demonstrate that caspase-11 is protective during intestinal carcinogenesis.

### Increased angiogenesis and reduced cell death characterise the enhanced carcinogenesis phenotype of Casp11^−/−^ mice

**Fig. 2:**
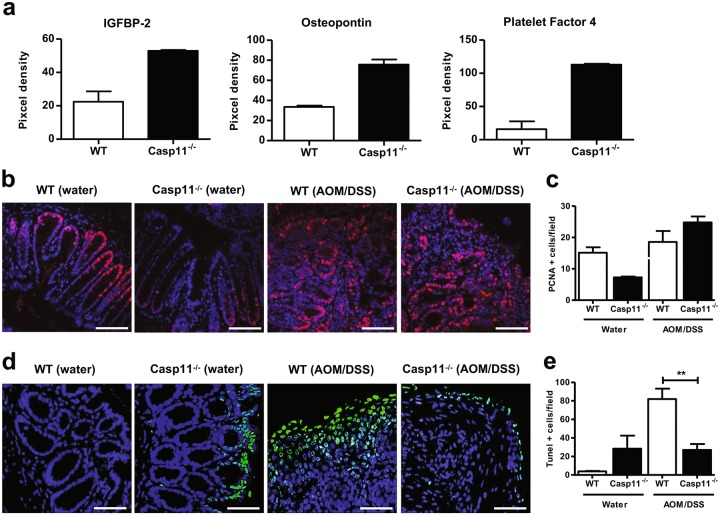
Increased angiogenesis and reduced cell death characterise the enhanced CAC phenotype of *Casp11*^-/-^ mice. **a** Semi-quantitative IGFBP-2, osteopontin and platelet factor 4 expression in AOM/DSS-treated WT and *Casp11*^−/−^ colon homogenates 105 days post initial AOM IP injection, as determined using an angiogenesis proteome profiler array and ImageJ densitometry. Data represent mean ± SEM of *n* = 2 AOM/DSS-treated mice for both groups. **b**–**e** Representative immunofluorescent images of proliferating cell nuclear antigen (PCNA) (**b**) and TUNEL (**d**) positive epithelial cells in distal colon tissue sections of AOM/DSS-treated WT and *Casp11*^−/−^ mice (scale bars = 50 μm (**b**) and 500 μm (**d**)). **c**, **e** Quantification of PCNA (**c**) and TUNEL (**e**) positive epithelial cells shown in **b** and **d**

Angiogenesis is a pre-requisite for tumour invasion and inhibition/depletion of caspase-1 has been implicated in the improvement of angiogenesis and blood supply in functional angiogenesis murine models [29, 30]. As substantially higher levels of invasive tumours were observed in AOM-DSS-treated *Casp11*^−/−^ colons, compared to WT (Fig. 1g, h), we looked for differences in expression of angiogenesis-related proteins in colons from the two treatment groups. An angiogenesis proteome profile array revealed enhanced expression of the proteins IGFBP-2, osteopontin (OPN) and platelet factor 4 (PF4) in *Casp11*^−/−^ homogenates (Fig. 2a, Supplementary Figure 2). OPN expression has been previously shown to enhance colon cancer cell growth, invasion and angiogenesis [31], and both IGFBP-2 and PF4 have been identified as biomarkers for the early detection of colorectal cancer [32, 33]. These findings suggest that caspase-11 regulates the expression of proteins associated with early stage angiogenesis during CRC, and strengthen the evidence for a protective role for caspase-11 during colorectal cancer.

The colonic mucosa of individuals with colon carcinoma is hyper-proliferative when compared with that from normal individuals [34]. To determine the consequence of caspase-11 deficiency on intestinal epithelial cell (IEC) proliferation during established CAC, proliferating cell nuclear antigen (PCNA) staining was assessed in distal colon sections from CAC in WT and *Casp11*^−/−^ mice. Results show a moderate, although not significant, increase in PCNA-positive IECs in *Casp11*^−/−^ colon sections (Fig. 2b, c). Consistent with our previous observations [12], less basal intestinal cell proliferation was observed in untreated *Casp11*^−/−^ colons, compared to WT controls (Fig. 2b, c). We also find that overall proliferation rates in healthy WT murine colons are similar to those of AOM-DSS-treated WT colons (Fig. 2c), which has been observed by other groups [4, 27, 35]. This may be explained by the fact that rapid proliferation of Lgr5^+^ stem cell-generated transit amplifying (TA) cells occurs in the lower half of crypts in healthy WT colons (Fig. 2b) [36]. However, DSS causes mucosal damage, destroying Lgr5^+^ cells and altering normal proliferation rates [37], while transformed IECs emerge as the rapidly proliferating population.

Given the key role of caspase-11 in driving pyroptotic and other cell death pathways [8, 9, 38, 39], we addressed whether the increased tumourigenic load observed in AOM-DSS-treated *Casp11*^−/−^ mice could be explained by reduced levels of IEC death. Both pyroptotic and apoptotic modes of cell death are detectable by TUNEL staining [40]. Distal colon sections of AOM-DSS-treated mice revealed a significantly lower number of TUNEL-positive IECs in *Casp11*^−/−^ compared to WT colons, confirming the importance of caspase-11 in promoting cell death during advanced stages of CAC (Fig. 2d, e).

### Reduced IL-1β production in Casp11^−/−^ mice during advanced stages of CAC

**Fig. 3:**
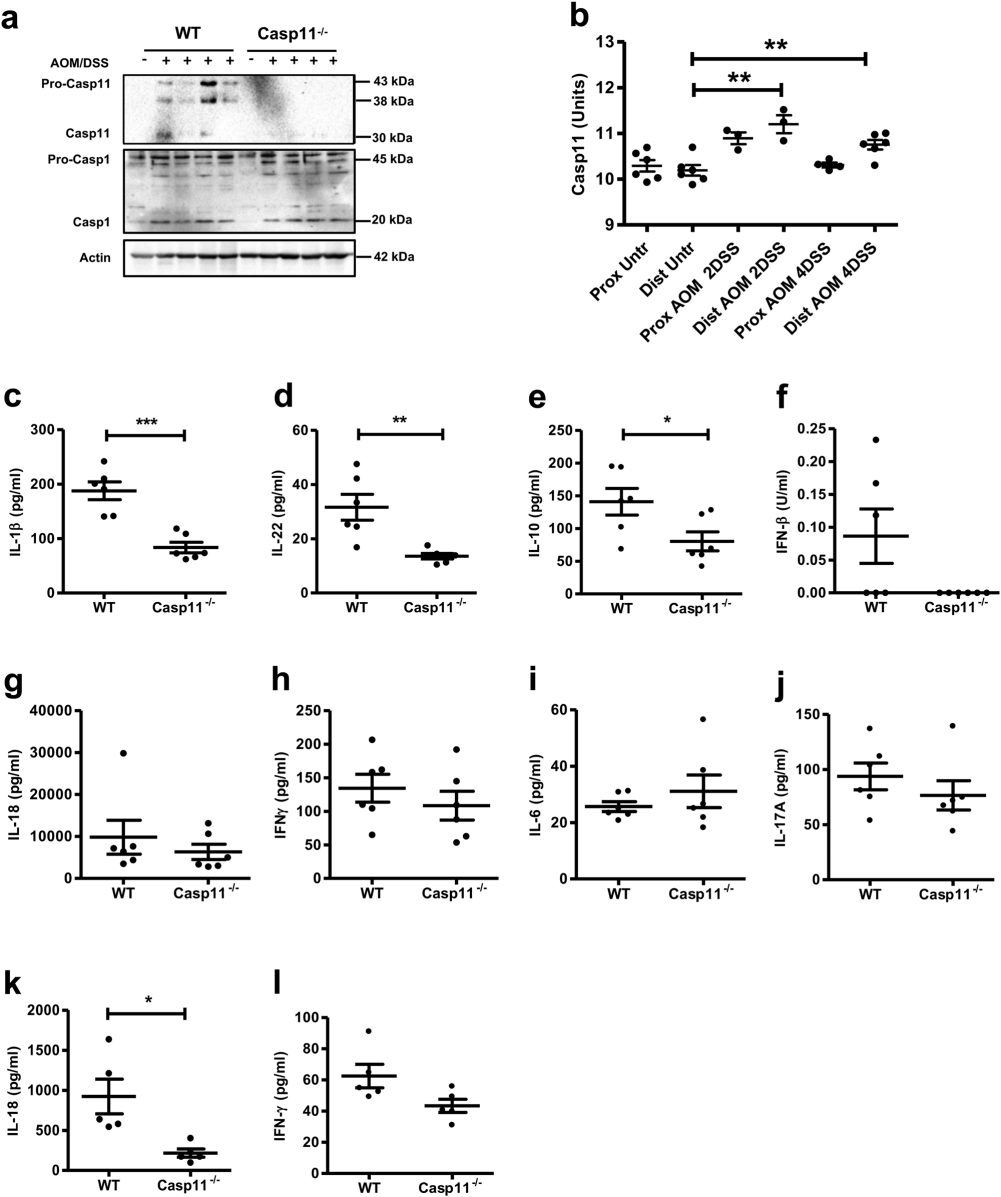
Reduced IL-1β production in *Casp11*^-/-^ mice during advanced stages of CAC. **a** Western blot analysis of caspase-11 and caspase-1 expression in colon homogenates from AOM/DSS-treated WT and *Casp11*^−/−^ mice on day 105 post initial AOM IP injection. Each lane represents an individual mouse. **b** Analysis of relative Casp11 mRNA expression in AOM/DSS-treated C57BL/6 J colon tissue using the published dataset GSE64658. Data represent mean ± SEM; *p *<* 0.05; **p *<* 0.01; (two-tailed independent Student *t-*test). **c**–**l** Cytokine levels in colon homogenates of AOM/DSS-treated WT and *Casp11*^−/−^ mice on day 105 (**c**–**j**) and day 21 (**k**, **l**) post initial AOM IP injection, as measured by ELISA. Data represent mean ± SEM of *n* = 6 (**c**–**j**) and *n* = 5 (**k**, **l**) AOM/DSS-treated mice for both groups; *p *<* 0.05; **p *<* 0.01; ***p *<* 0.001 (two-tailed independent Student *t-*test)

Colon homogenates from AOM-DSS-treated WT mice demonstrate elevated caspase-11 expression at the 15 week experimental endpoint (Fig. 3a). This is in agreement with the enhanced expression of human caspase 4 and 5 during CRC [19], strongly suggesting an active involvement of inflammatory caspases during CAC. As canonical inflammasome activity is not affected by caspase-11 deficiency [12], we expect that a certain level of caspase-1 activation will still occur in AOM-DSS-treated *Casp11*^−/−^ colons. Fig. 3a shows that similar levels of full length and processed (p20) caspase-1 subunits are apparent in both WT and *Casp11*^−/−^ colon homogenates. However, conclusions regarding caspase-1 activity cannot be made from this result, as a recent report suggests that caspase-1 immunoblots are not representative of its activity, and that processed caspase-1 subunits (p20/p10—previously considered to be active), in fact represent inactivated caspase-1 subunits [41]. Consistent with our findings, mRNA analysis from an independent AOM-DSS trial [42] also demonstrates significantly elevated caspase-11 mRNA levels in distal colons following both two and four rounds of DSS treatment (Fig. 3b).

Cytokine expression was quantified by ELISA in WT vs. *Casp11*^−/−^ mice during CAC, using colon homogenates from 15 week endpoints. Data demonstrate that colons from *Casp11*^−/−^ mice have significantly less IL-1β, IL-22 and IL-10 than their WT littermate controls (Fig. 3c–e). As the tumour load was higher in *Casp11*^−/−^ colons, compared to WT, it is possible that enhanced tumorigenesis may contribute to the cytokine differences observed between genotypes. However, defective IL-1β production during CAC in *Casp11*^−/−^ mice is consistent with previous studies evaluating inflammasome signalling during CAC, including *Asc*^−/−^, *Casp1*^−/−^*/11*^−/−^ and *Nlrp1*^−/−^ mice [20, 25]. The specificity of these data are demonstrated by the observation that no significant differences in colon levels of IFNβ IL-18, IL-1α IFNγ, IL-6 or IL-17a were detected at this advanced stage of CAC (Fig. 3f–j). Importantly, decreased IL-18, and to a lesser extent IFNγ, production was evident in *Casp11*^−/−^ mice at 2 weeks (Fig. 3k, l), which may be ultimately responsible for defective IL-22 production observed in these mice during both experimental colitis and CAC [43]. Thus, during advanced stages of CAC, intestinal IL-1β and IL-22 appear to be the cytokines most significantly affected by caspase-11 deficiency.

### Caspase-11 is required for STAT1 activation during CAC

**Fig. 4:**
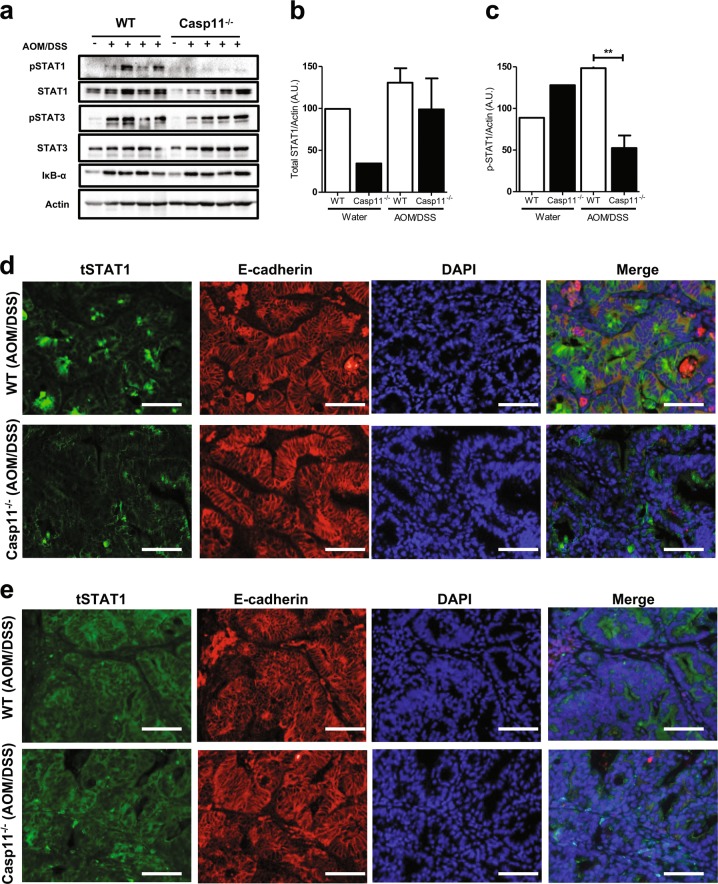
Caspase-11 mediates STAT1 activation during CAC. **a** Western blot analysis of phosho-/total STAT1, STAT3, IκBα, and actin (loading control) in colon homogenates from AOM/DSS-treated WT and *Casp11*^−/−^ mice on day 105 post initial AOM IP injection. Each lane represents an individual mouse. **b** Total STAT1 and **c** pSTAT1 expression densitometry (relative to actin) in colon homogenates from AOM/DSS-treated WT and *Casp11*^−/−^ mice. **p *<* 0.01 (two-tailed unpaired t-test). Representative immunofluorescent images of **d** pSTAT1; and **e** total STAT1; co-stained for the epithelial marker E-cadherin (red) and DAPI (blue) in distal colon sections from AOM/DSS-treated WT and *Casp11*^−/−^ mice at day 105 (scale bar = 20 μm)

We have previously shown that STAT1 expression and phosphorylation levels are upregulated during DSS-colitis [12]. STAT1 activation is also a consequence of CAC, and has been proposed as a mechanism for the protective effects of the NLRP3 inflammasome during CAC [4]. We therefore investigated the expression and activation status of STAT1 in AOM-DSS-treated WT and *Casp11*^−/−^ colon homogenates, and observed a marked impairment of STAT1 activation in *Casp11*^−/−^ colons of CAC-treated mice (Fig. 4a–c). In contrast, caspase-11-specific alterations were not observed in total or phosphorylated STAT3, or IκB expression levels (Fig. 4a). IF staining of WT colon tumour tissue indicated STAT1 activation in IECs during AOM-DSS treatment, and confirmed the STAT1 activation deficiency in *Casp11*^−/−^ colon tumours (Fig. 4d). Total STAT1 levels appear to be similar in tumour tissue from WT and *Casp11*^−/−^ colons (Fig. 4e). STAT1 activation appears to be occurring predominantly in tumorigenic epithelial cells, as F4/80-positive immune cells did not express phosho-STAT1 (Supplementary Figure 3). These results suggest that the protective role of caspase-11 during experimental CAC is mediated by anti-tumorigenic STAT1 signalling in IECs.

### Impaired STAT1 activation and increased IEC proliferation associates with caspase-11 deficiency during development of CAC

**Fig. 5:**
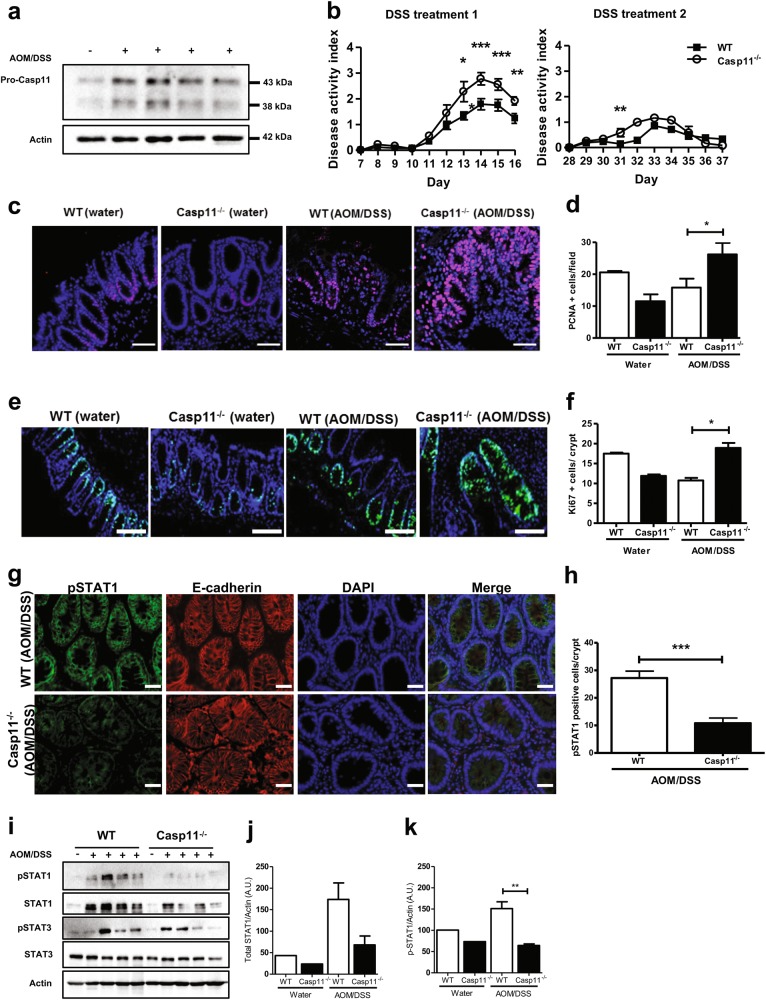
Impaired STAT1 activation and increased IEC proliferation are evident in *Casp11*^-/-^ mice during CAC development. **a** Western blot revealing caspase-11 expression in colon homogenates of C57BL/6 mice on day 42 post initial AOM IP injection. Each lane represents an individual mouse. **b** Disease activity index during each DSS administration. Data represent mean ± SEM of *n* = 6 (WT) and *n* = 5 (*Casp11*^−/−^) AOM/DSS-treated mice; *p *<* 0.05; **p *<* 0.01; ***p < 0.001 (two-way ANOVA followed by the Bonferroni post test). **c**–**f** Representative immunofluorescent images of proliferating cell nuclear antigen (PCNA) (**c**); and Ki67 (**e**); positive epithelial cells in distal colon tissue sections of AOM/DSS-treated WT and *Casp11*^−/−^ mice (scale bars = 50 μm, **c** and 100 μm **e**). Quantification of PCNA (**d**) and Ki67 (**f**) positive epithelial cells shown in **d** and **f**. Data represent mean ± SEM of *n* = 6 (WT) and *n* = 5 (*Casp11*^−/−^) AOM/DSS-treated mice; *p *<* 0.05 (two-tailed independent Student *t-*test). **g** Representative IF images of distal colon sections from AOM/DSS-treated WT and *Casp11*^−/−^ mice at day 42, stained for pSTAT1, epithelial marker E-cadherin and DAPI (Scale bar = 20 μm). **h** Quantification of pSTAT1 positive IECs cells in IF stained colon sections. (**i**) Western blot analysis of STAT1 and STAT3 activation in colon homogenates from AOM/DSS-treated WT and *Casp11*^−/−^ mice on day 42 post initial AOM IP injection. Each lane represents an individual mouse. Expression dfensitometry (relative to actin) of **j** total STAT1 and **k** pSTAT1; in colon homogenates from AOM/DSS-treated mice on day 42 post initial AOM IP injection. **p *<* 0.01 (two-tailed unpaired *t*-test)

To further explore the association between caspase-11 and STAT1 signalling during CAC, we examined whether deficiencies in STAT1 activation also occurred at earlier stages of CAC. To address this experimentally, we terminated the AOM-DSS protocol (Fig. 1a) at 6 weeks, a stage at which adenomas were developing, but were not yet macroscopically detectable. Western blot analysis of colon homogenates showed increased expression of caspase-11 at this stage of CAC development (Fig. 5a). As observed in the 15 week AOM-DSS trial, *Casp11*^−/−^ mice displayed higher disease activity scores than their WT littermates during DSS treatments (Fig. 5b, Supplementary Figure 4). Symptom variability can occur between DSS treatments [37], and we observed a high overall disease activity in DSS treatment 1 of this trial (Fig. 5b), due to increased detection of blood in the faecal pellets of *Casp11*^−/−^ mice (Supplementary Figure 4c).

Analysis of WT and *Casp11*^−/−^ colons at the 6 week tumour development stage revealed significantly increased IEC proliferation in *Casp11*^−/−^ colons compared with treatment matched WT colons, as assessed by staining and histological evaluation of the proliferation markers PCNA (Fig. 5c, d) and Ki67 (Fig. 5e, f). In contrast to 15 week colons, TUNEL staining revealed no significant differences in the levels of IEC death between WT and *Casp11*^−/−^ colons at 6 weeks (Supplementary Figure 5a, b). Alterations in angiogenic markers were not observed at the tumour development stage (Supplementary Figure 5c, d).

Total and phospho-STAT1 expression levels were also assessed, to determine whether the anti-proliferative role for caspase-11 observed in IECs during CAC development was consistent with the anti-proliferative function of STAT1. IF staining in dysplastic colon tissue showed that less STAT1 activation occurred during tumour development in *Casp11*^−/−^ mice, compared to WT (Fig. 5 g, h; Supplementary Figure 6). Immunoblot results confirm this observation, revealing that STAT1 activation is also caspase-11 dependent at the tumour development stage (Fig. 5i-k). Total STAT1 expression analysis shows that there is also a trend towards lower total STAT1 levels in *Casp11*^−/−^ colons (Fig. 5i, j). This observation is not surprising, as STAT1 activation has been shown to positively regulate its own expression [44]. These data support the hypothesis that caspase-11 mediates anti-tumourigenic STAT1 activity during experimental CAC.

### STAT1 activation in primary murine macrophages is regulated by caspase-11

**Fig. 6:**
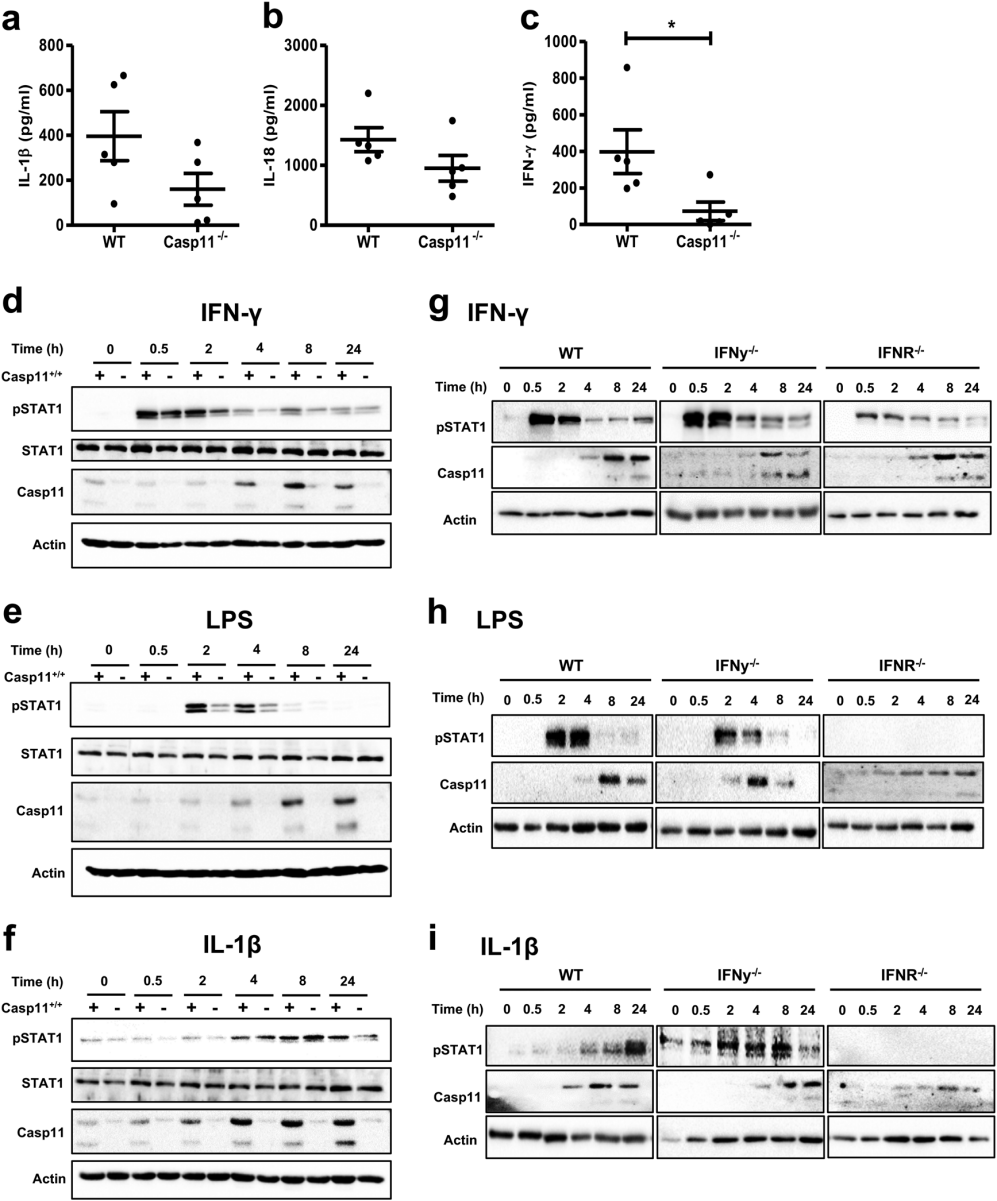
Caspase-11 regulates STAT1 activation in primary murine macrophages. **a**–**c** Distal colon explant tissue isolated from AOM/DSS-treated WT and *Casp11*^−/−^ mice on day 21 post initial AOM IP injection were cultured for 20 h. Supernatants were analysed for the production of IL-1β (**a**), IL-18 (**b**), and IFN-γ (**c**), by ELISA; *p *<* 0.05 (two-tailed independent Student *t-*test). **d**–**f** Western blot analysis of pSTAT1, total STAT1 and caspase-11 expression in WT and *Casp11*^−/−^ BMDMs treated with IFN-γ (**d**), LPS (**e**) and IL-1β (**f**). **g**–**i** Western blot analysis of STAT1 and caspase-11 expression in WT, *Ifnar*^−/−^ and *Ifn-γ*^−/−^ BMDMs treated with IFN-γ (**g**), LPS (**h**) and IL-1β (**i**). Blots are representative of more than three independent experiments

The findings presented in this study identify IL-1β and STAT1 as the key cytokine and transcription factor, respectively, affected by caspase-11 deficiency during established CAC. The defective IL-1β production observed suggest a role for a functional non-canonical inflammasome during CAC. However, a link between non-canonical inflammasome activation and STAT1 signalling pathways is not well characterised. IFNγ represents an obvious link between STAT1 and caspase-11, as it drives STAT1-dependent caspase-11 expression [45]. No significant differences in IFNγ levels were detected in *Casp11*^−/−^ colon homogenates at the 6 or 15 week stages of CAC development (Fig. 3h, l), however *Casp11*^−/−^ colon explants taken 3 weeks into the AOM-DSS protocol secreted significantly less IFNγ than their WT counterparts (Fig. 6c). Although impaired STAT1 activation appears to occur predominantly in *Casp11*^−/−^ IECs during colorectal tumorigenesis, we sought to determine whether caspase-11 also has the ability to regulate STAT1 activity in primary macrophages. Primary bone marrow derived macrophages (BMDM) from WT and *Casp11*^−/−^ mice were stimulated with IFNγ, LPS, or IL-1β and monitored for Phospho-STAT1 over 24 h (Fig. 6d–f). As expected, STAT1 phosphorylation was induced within 30 min of IFNγ stimulation in both WT and *Casp11*^−/−^ BMDM, and caspase-11 was upregulated within 4 h in WT BMDM only (Fig. 6d). Phospho-STAT1 levels decreased after 2 h but a moderate, sustained expression remained up to 24 h. This STAT1 activity was reduced in *Casp11*^−/−^ BMDM, suggesting that sustained IFNγ-driven STAT1 activity is partially dependent on caspase-11 (Fig. 6d). LPS stimulation resulted in significant STAT1 activation within 2 h, which decreased by 8 h. Similar to the effect of IFNγ, LPS-induced STAT1 activation was impaired in *Casp11*^−/−^ BMDM, supporting a role for caspase-11 in the regulation of this STAT1 activation pathway (Fig. 6e). Data shown in Fig. 6f demonstrate that IL-1β stimulated BMDM significantly upregulate caspase-11 within 2 h, which is sustained until 24 h. Phospho-STAT1 was detected within 4 h IL-1β stimulation in both WT and *Casp11*^−/−^ BMDM, although no significant caspase-11 dependency for STAT1 activity was observed (Fig. 6f). Stimulations were also performed in BMDM from WT, *Ifnar*- and *Ifnγ*-deficient mice, to determine whether LPS and IL-1β-stimulated activation of STAT1 were being mediated via Type I or II IFN signalling (Fig. 6g-i). Fig. 6g confirms that IFNγ-stimulation results in STAT1 activation in both *Ifnar*^−/−^ and *Ifn*γ^−/−^ BMDM. Consistent with previous reports [12, 15], LPS-mediated induction of caspase-11 was significantly attenuated in *Ifnar*^−/−^ BMDM (Fig. 6h). Similarly, STAT1 activation was unaffected in *Ifnγ*^−/−^ BMDM, but completely inhibited in *Ifnar*^−/−^ BMDM, confirming that LPS-mediated STAT1 activation occurs via Type I IFNs (Fig. 6h). Analogous to LPS stimulations, IL-1β-mediated upregulation of caspase-11 and STAT1 activation require type I IFN signalling (Fig. 6i). These experiments demonstrate that both LPS and IL-1β stimulate caspase-11 expression and STAT1 activation in BMDM via type I IFNs, and reveal a role for caspase-11 in LPS-mediated STAT1 activation in BMDM.

### Caspase-11 regulates LPS- and IL-1β-mediated STAT1 activation in IECs

**Fig. 7:**
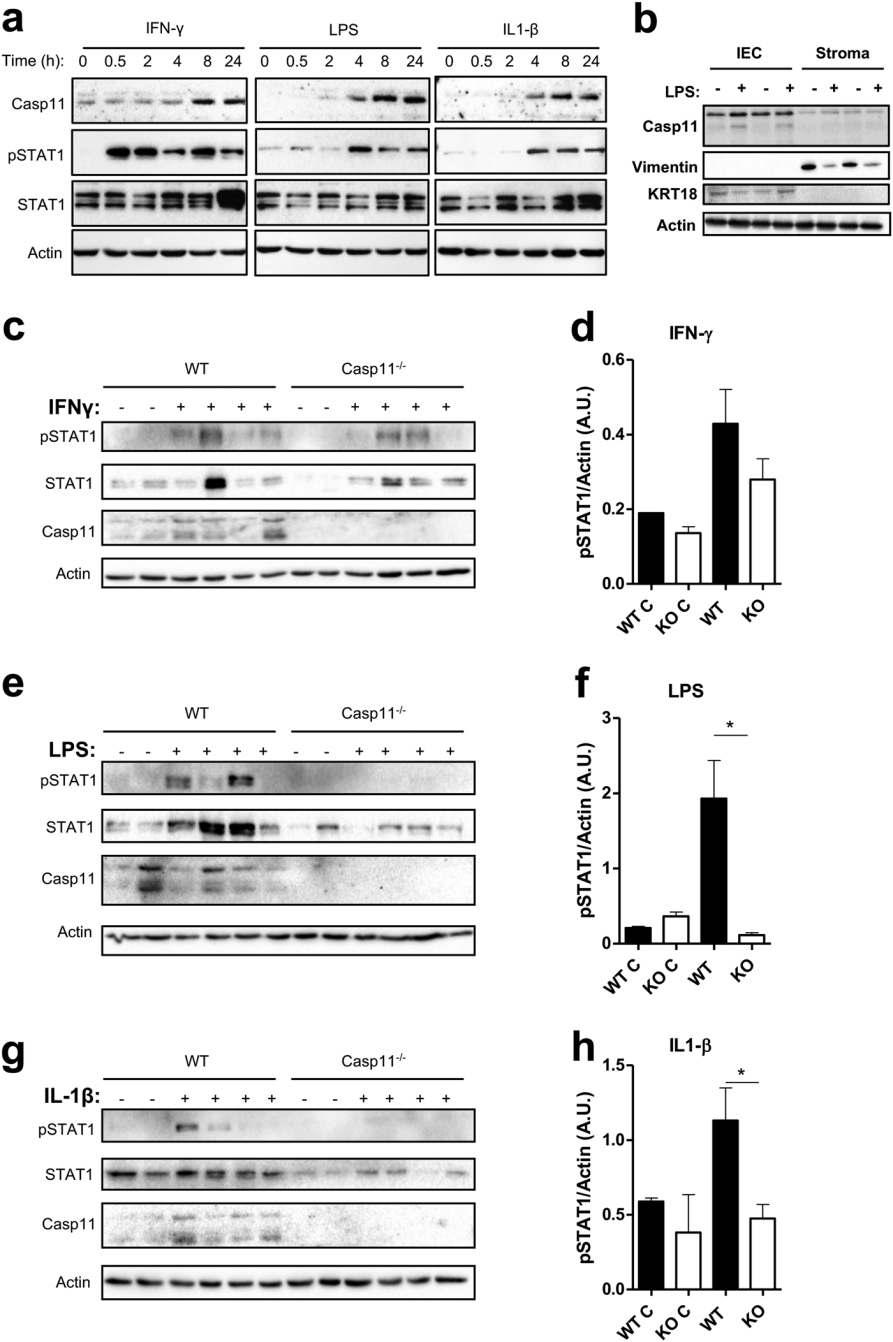
Caspase-11 regulates LPS and IL-1β-mediated STAT1 activation in IECs. **a** Western blot timecourse analysis of caspase-11, pSTAT1 and total STAT1 expression in cell lysates from CT26 colon cancer cells stimulated with IFN-γ, LPS and IL-1β. **b** Representative western blot showing the relative expression levels of caspase-11, vimentin (mesenchymal cell marker) and keratin 18 (epithelial cell marker) in IECs and mesenchymal cell lysates isolated from WT colon tissue explants, which had been incubated in media or with LPS for 20 h. **c**, **e**, **g** WT and *Casp11*^−/−^ colon explant tissue were left untreated (*n* = 2) or treated (*n* = 4) with IFN-γ (**c**), LPS (**e**), and IL-1β (**g**). Following 20 h incubation, purified IEC lysates were analysed for expression of caspase-11, pSTAT1 and total STAT1 by western blot. **d**, **f**, **h** Densitometric analysis of pSTAT1 (relative to actin) in WT and *Casp11*^−/−^ IEC lysates following stimulation with IFN-γ (**d**), LPS (**f**), and IL-1β (**h**). Data represent mean ± SEM of *n* = 4 (untreated) and *n* = 8 (treated) WT and *Casp11*^−/−^ mice; *p *<* 0.05; **p *<* 0.01 (two-tailed independent Student *t-*test)

To provide further evidence of a role for caspase-11 during CAC, we investigated whether stimulation with IFNγ, LPS and IL-1β was capable of upregulating caspase-11 in an established murine CRC cell line, CT26. The data shown in Fig. 7a confirm that CT26 cells respond to all stimuli by upregulating caspase-11. Interestingly, the kinetics of caspase-11 upregulation following LPS and IL-1β stimulation closely correlate with those of STAT1 activation, supporting the notion that indirect STAT1 activation requires caspase-11 in colorectal carcinoma cells.

Having established a role for caspase-11 in STAT1 activity during CAC, and a link between IL-1β and STAT1 signalling in BMDM, we examined whether caspase-11 could also influence LPS or IL-1β-mediated STAT1 activation in IECs. To establish a system whereby primary IECs can be stimulated in vitro, colon explant tissue (1 cm sections) were incubated in media (alone or with stimulant) for 20 h before separating the IEC from the lamina propria (i.p.). The purity of IECs was confirmed by probing for cytokeratin-18, and the lamina propria cells by the presence of vimentin. Furthermore, LPS stimulation reveals that caspase-11 is selectively upregulated by IECs (Fig. 7b). To determine whether caspase-11 could regulate STAT1 activation pathways in IECs, colon explants from WT and *Casp11*^−/−^ mice were stimulated with IFNγ, LPS and IL-1β before purifying IECs. Western blot revealed that STAT1 activation was similar in WT and *Casp11*^−/−^ cells following IFNγ stimulation (Fig. 7c, d), confirming that caspase-11 cannot influence direct STAT1 activation via IFNγ. In contrast, both LPS and IL-1β-mediated activation of STAT1 was impaired in *Casp11*^−/−^ IECs (Fig. 7e–h), suggesting that caspase-11 regulates crosstalk between LPS/IL-1β-mediated inflammation and STAT1 activation pathways in IECs.

**Fig. 8:**
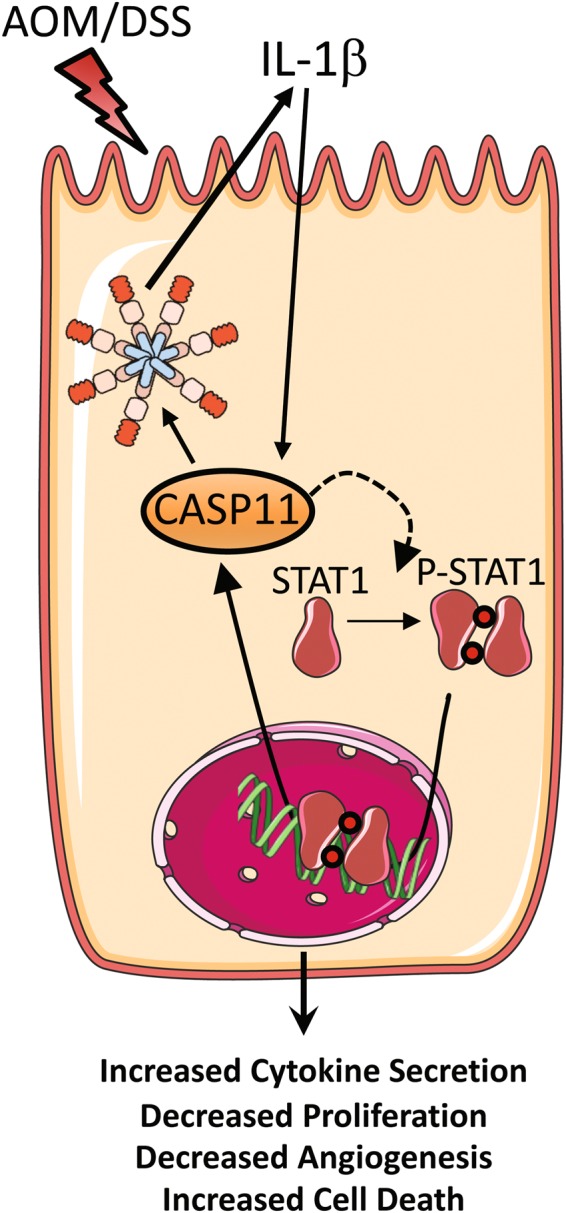
Graphical representation—the anti-tumorigenic role of caspase-11 during CAC. Caspase-11 has dual roles during AOM-DSS-induced colitis-associated carcinogenesis: (i) enhancing IL-1β production during tumorigenesis; and (ii) regulating STAT1 activation in IECs in response to IL-1β. The effects of caspase-11 are anti-tumorigenic in this system, causing decreased angiogenesis and increased IEC death

Figure 8 provides a summary of the novel findings presented in this manuscript: a suppressive role for caspase-11 during colorectal carcinogenesis; the ability of IL-1β to induce caspase-11 upregulation; and a requirement for caspase-11 during IL-1β-mediated STAT1 activation in IECs.

## Discussion

This study reveals that the anti-tumorigenic effects of STAT1 during CAC are critically dependent upon caspase-11. At advanced CAC stages, we show that *Casp11*^−/−^ mice have more aggressive and higher incident tumours than their WT littermates. A previous study on CAC has reported no observable differences between WT and *Casp11*^−/−^ mice [17], however the study performed a shorter trial (8.5 week) and observed much fewer adenomas/colon (0.5–1.5 compared with 4–15 in our study), suggesting that differences may have become apparent if the model had been extended.

Consistent with the enhanced tumorigenesis observed in *Casp11*^−/−^ mice, we observe defective STAT1 activation in caspase-11 deficient AOM-DSS-treated mice at both advanced (15 week) and progression (6 week) stages of CAC. Increased angiogenic markers and decreased IEC death were also observed in *Casp11*^−/−^ tumours, processes which are usually inhibited by STAT1 [46]. There is significant evidence for the anti-tumorigenic role of STAT1 in both human and murine studies, as STAT1 is believed to be responsible for the anti-proliferative effects of IFN [47]. Studies in patients have suggested a negative correlation between STAT1 and CRC tumorigenesis and tumour cell proliferation [43, 48], and STAT1 has been proposed as a biomarker for good prognosis in CRC [49]. Whether, similar to murine caspase-11, human caspases-4 and -5 regulate STAT1 activity during CRC progression in patients has yet to be determined. In support of our findings, *Stat1*^−/−^ mice have increased susceptibility to experimentally-induced tumours and spontaneously develop mammary adenocarcinomas [50, 51]. Furthermore, a recent study has shown that mice with specific deletion of STAT1 in intestinal epithelial cells (STAT1-DIEC) are more susceptible to AOM-DSS-induced CRC, exhibiting higher grade adenomas than their similarly treated littermate controls [52]. Interestingly, this study found that male STAT1-DIEC mice were significantly more susceptible than their female counterparts, suggesting that, in epithelial compartments, the tumour suppressor function of STAT1 may be influenced by gender [52].

Although we reveal a novel link between the non-canonical inflammasome and STAT1 activity during CAC, components of the canonical inflammasome (NLRP3 and caspase-1), have been previously proposed to mediate their protective effects during CAC via IL-18-mediated IFNγ-STAT1 signalling [4]. In agreement with this, our study shows that at early stages (3 weeks) of CAC development, reduced IL-18 levels were measured in *Casp11*^−/−^ colon homogenates (compared to their WT littermates), which correlated with less IFNγ secretion from *Casp11*^−/−^ colon explants. However, at advanced stages of CAC we show that IL-1β levels are more significantly affected than IL-18 levels in *Casp11*^−/−^ colons. These results suggest that caspase-11-mediated IL-1β signalling is protective during experimental CAC. Although high levels of IL-1β have been associated with tumour progression [53], a recent study has suggested that physiological levels of IL-1 are essential for antitumor immunity [54]. For example, IL-1β is required for the polarisation of IFNγ-producing CD8^+^ T cells; and is also implicated in the immunostimulatory properties of chemotherapy in the generation of anti-tumour γδT cells [55, 56]. In support of our finding, Allen et al. [20] also show impaired IL-1β levels in *Asc*^−/−^ and *Casp1*^−/−^ mice, correlating with increased tumour burden, following 8 weeks AOM-DSS administration.

There are tissue and cell-specific differences in the regulation of caspase-11; while type I IFNs are strongly required for caspase-11 activation in BMDM [15, 57], *Ifnar*^−/−^ and *Trif*^−/−^ mice show no impairment in caspase-11 transcription or activation in the murine colon during DSS-colitis [12]. Previous data show that IFNγ-mediated STAT1 signalling is a key requirement for intestinal caspase-11 upregulation and activation in IECs during DSS-colitis [12]. The current study demonstrates that caspase-11 is strongly induced in both BMDM and IECs following IL-1β stimulation, which has not been previously reported. As IL-1β activates NFκB via IL-1 receptor binding and MyD88 signalling, and caspase-11 contains κB elements within its promoter region [45], it is not surprising that IL-1β is capable of driving caspase-11 transcription. Therefore, in addition to elucidating a critical role for caspase-11 in IL-1β production in established colorectal tumours, our data show that, once initiated, IL-1β signalling drives caspase-11 upregulation in BMDM, IECs and CT26 murine CRC cells, to further promote its pro-inflammatory effects. Whether IL-1β has similar effects on caspase-11 in other cell types has yet to be determined.

This is the first report, to our knowledge, that demonstrates a positive relationship between IL-1β signalling and STAT1 activation, which is regulated by caspase-11. We show that caspase-11 is required for IL-1β- and LPS-driven STAT1 activation in IECs, suggesting that caspase-11 facilitates crosstalk between MyD88 and STAT1 signalling pathways at the intestinal barrier, to promote anti-tumorigenic signalling during inflammation-associated intestinal disease. A protective role for MyD88 during AOM-DSS-induced intestinal carcinogenesis has been previously reported [58]. Impaired induction of IFN-dependent genes were shown in AOM-DSS-treated colons of MyD88^−/−^ compared to WT mice, which could only be partially explained by defective IL-18 signalling, suggesting that additional TLR/IL-1 signalling pathways, such as IL-1β, may contribute to the protective effect of MyD88 during CAC [58]. Although we demonstrate a regulatory role for caspase-11 during LPS and IL-1β-mediated STAT1 activation in IECs, LPS-stimulated, but not IL-1βstimulated, primary macrophages from *Casp11*^−/−^ mice show impaired STAT1 activation. Thus, similar to the differential regulation of caspase-11 expression in IECs and BMDM [12], the regulatory role of caspase-11 may also differ between innate immune and epithelial cell types. Both LPS- and IL-1β-mediated STAT1 activation in BMDM are type I IFN-dependent, however the caspase-11 regulated mechanisms governing STAT1 activation in IECs have yet to be confirmed. Further studies are required to elucidate the mechanistic basis responsible for caspase-11-mediated STAT1 activation. Preliminary data suggest that the levels of negative STAT1 regulators, including SOCS1, are not affected by caspase-11 deficiency (J. Manils, E. Creagh, unpublished). We hypothesise that upregulated caspase-11 is capable of mediating STAT1 activation, either directly via its CARD domain or by the recruitment of another cytosolic molecule. Evidence for this hypothesis comes from previous studies which show a biphasic regulation of STAT1 in response to dsRNA, with an initial IFN-dependent phase followed by a STAT1 phosphorylation phase that is IFN-independent [59, 60]. The CARD domain of RIG-I can induce STAT1 activation independently of Type I IFN, and it has been proposed that a cytosolic CARD-containing molecule may be capable of STAT1 activation [59]. Overexpression studies suggest that IFN responses are amplified by RIG-I, in the absence of viral infection, via direct interaction of the RIG-I CARD region with the STAT1 SH2-transactivation domain, impeding STAT1 dephosphorylation and thereby sustaining its activation [61]. Whether caspase-11 is capable of STAT1 regulation in a manner analogous to that of RIG-I is currently under investigation.

STAT1 has a complex role during tumourigenesis, and it has been proposed to link intestinal inflammation and colon cancer by its involvement in crosstalk between stromal and epithelial cell populations [62]. Our data support the hypothesis that not all IL-1β signalling is involved in tumour promotion, as IL-1β is capable of stimulating caspase-11 expression, which can subsequently mediate STAT1 activation in IECs. This study identifies dual functions for caspase-11 during CAC—generating IL-1β via the non-canonical inflammasome; and mediating the tumour suppressor activities of STAT1.

## Materials and methods

### Mice

*Casp11*^−/−^ mice on the C57BL/6J background were obtained from J. Yuan (Harvard Medical School), and were further backcrossed with C57BL/6J mice for eight generations. Heterozygous breeding pairs were used to generate wild-type (WT) and *Casp11*^−/−^ littermates. Experiments were performed with 10–14-week-old female mice bred under specific pathogen-free conditions, under license and the approval of the local animal research ethics committee and the Irish Health Protection Regulatory Agency. The *Ifnar*^−/−^ and *Ifn-*γ^−/−^ mice, used to generate BMDM, were kindly provided by Prof. Ed Lavelle, TCD.

### Induction of colitis-associated CRC

Tumorigenesis in mice was induced by AOM (WAKO Chemicals) injection (intraperitoneally, 12.5 mg/kg) on day 0 and 21. On day 7, 28 and 49, 2% DSS (MP Biomedicals, MW 36 000–50 000) was supplemented in drinking water for 4 days. Animals were weighed and monitored daily for disease activity (weight loss, stool consistency and rectal bleeding (Scoring Table -Supplementary Figure 1a)). On day 105, mice were humanely killed and colons were removed from the ceco-colonic junction to the rectum. Experiment was performed three times with *n* = 6 mice/coded group (Power > 0.935; std dev 1.25; α error prob. 0.05: G-power software analysis). Each colon was processed as follows: A 0.5 cm section was taken from the distal and proximal (adjacent normal control) ends for histology and imaging; the next distal 1 cm colon tissue section was taken for western blot analysis; and the following 1 cm (distal-mid colon) section was used for cytokine measurement.

### Histology & Immunostaining

Following fixation (10% buffered formalin, 48 h), distal colon sections were processed, paraffin embedded, sectioned longitudinally (5 μm) and H&E stained. Histological assessment was performed by an experienced pathologist (E. Kay) in blinded fashion. For immunostaining, sections were deparaffinized in Histoclear (National Diagnostics) for 5 min and progressively rehydrated in decreasing concentrations of ethanol (100, 90 and 70%, 5 min), with final incubation in water. Antigen retrieval for Ki67 (LeicaBiosystems; NCL-Ki67p), phospho-STAT1 (Cell Signalling; 9167), total STAT1 (Cell Signalling; 9172) and E-Cadherin (eBiosciences; 50–3249–80) was carried out by boiling in Tris-EDTA, pH 9.0 (20 min), cooling to RT, 1 h blocking (PBS, 20% FBS) before incubation with the primary antibody (1 h, RT). For PCNA (Abcam), antigen retrieval was performed by 10 min incubation in boiling 0.01 M sodium citrate buffer (pH 6.0). Sections were washed (PBST) and incubated with primary antibody (1:100, o/n, 4 °C). Slides were washed and incubated with Alexa Fluor-conjugated secondary antibodies (1:500 dilution, Invitrogen) before mounting (DakoCytomation). Images were obtained using an Olympus BX51 microscope. Quantitative fluorescence intensity of PCNA-positive epithelial cells was analysed in three specifically selected areas of colonic tissue/mouse using Imaris software. For Ki67 and pSTAT1 quantifications, the number of positive cells/10 well-oriented crypts/mouse were analysed.

### TUNEL staining

Distal colonic tissue sections were analysed by fluorescence microscopy using an in situ cell death detection kit (Roche) according to the manufacturer’s protocol. ×20 images of stained tissue sections were taken using the Olympus BX51 microscope. Three random optical fields/colon from each mouse colon were chosen. Within each of the three fields/mouse, three separate areas (each containing ~100 cells) were enlarged and quantified for TUNEL positivity in control and AOM/DSS-treated animals.

### Cytokine measurements

Sections from tumorigenic distal-mid colon regions were homogenised in lysis buffer (PBS, 1% NP-40 and Protease-inhibitor cocktail (Roche)). Colon homogenates were centrifuged (20,000 × *g*, 10 min, 4 ^o^C) and supernatants normalised using the BCA method (ThermoFisher). For colon explants, colons were opened longitudinally, PBS washed and cut into 3 cm sections. Each section was cultured in media (DMEM + GlutaMAX, 10% FBS, 1% Pen-Strep) for 20 h. Supernatants were analysed for secreted cytokine levels using Biolegend (IL-1β, IL-10, IFN-γ, IL-6 and IL-17a, and IL-1α) and R&D Systems (IL-22) ELISA kits. IL-18 levels were determined using IL-18 capture (MBL; DO47–3) and detection (MBL; D048–6) antibodies. IFN-β levels were determined using IFN-β capture (Santa Cruz; sc57201) and detection (PBL Assay Science; 32400–1) antibodies.

### Colon explant stimulation and isolation of colonic epithelial cells

Colon sections (3 cm) were untreated or treated with 1 μg/ml LPS (Sigma; *E. coli* 0111.B4); 20 ng/ml murine IFNγ (Peprotech; 315–05); or 20 ng/ml murine IL-1β (R&D; 401-ML-005) for 24 h. Sections were agitated (4 °C, 2 h) in 0.5 ml Cell Recovery Solution (BD). Crypts were dislodged by vigorous vortex and separated from the lamina propria fraction by aspiration. Fractions were centrifuged at (350 × *g*, 10 min), pellets lysed (100 μl ice-cold RIPA buffer) and protein concentration was calculated (BCA method) before immunoblotting.

### Cell culture and stimulation

The CT26 cell line was sourced from ATCC. Bone marrow derived macrophages (BMDMs) from WT, Casp11^−/−^, IFNAR^−/−^ and IFN-γ^−/−^ mice were cultured (DMEM, 10% FBS, 20% L929-medium) for 9–11 days, plated at 3 × 10^5^ cells/well in a 24 well plate and cultured o/n. BMDM stimulations with LPS, IL-1β and IFNγ were performed at the same concentrations used for colon explant stimulations.

### Immunoblotting

Colon tissue was homogenised in RIPA buffer, clarified and normalised using the BCA method. 20 μg protein was run on 10–12% SDS-PAGE gels, transferred to nitrocellulose and probed with primary antibodies against caspase-11 (Sigma; C1354), caspase-1 (Santa Cruz; sc-514), IL-1β (Santa Cruz; sc7884), STAT1 (Cell Signalling; 9172), phospho-STAT1 (Cell Signalling; 7649), STAT3 (Cell Signalling; 8768), phospho-STAT3 (Cell Signalling; 9131), β-actin (AC-15, Sigma; A3854), Cytokeratin-18 (Santa Cruz; sc-31700) or Vimentin (abcam; EPR3776), followed by incubation with the appropriate HRP-secondary antibody.

### Angiogenesis proteome array

Angiogenic proteins were detected in colon homogenates using an Angiogenesis proteome profiler array (R&D Systems; ARY015) according to manufacturer’s protocol.

### Statistical analysis

Data were analysed using GraphPad Prism 5 software. Unpaired two-tailed Student *t*-tests were used to compare the mean values between two groups. Statistical differences in mean values between more than two experimental groups were determined by two-way analysis of variance (ANOVA) followed by Bonferroni post test. *p* values < 0.05 were considered statistically significant.

## Supplementary information

Supplementary Figure 1

Supplementary Figure 2

Supplementary Figure 3

Supplementary Figure 4

Supplementary Figure 5

Supplementary Figure 6
