# Supplementary material for: Caspase-11 regulates the tumour suppressor function of STAT1 in a murine model of colitis-associated carcinogenesis
Source: Oncogene. 2018 Dec 11;38(14):2658–74. doi: 10.1038/s41388-018-0613-5 (PMC6484510; doi:10.1038/s41388-018-0613-5)
Supplement: Supplementary file 1 — Supplementary Figure 1 [file 41388_2018_613_MOESM1_ESM.pdf]

a

Score	Weight loss (%)	Stool consistency (Diarrhea score)	Occult/Gross bleeding* (Bleeding score)
0	None	Well formed pellets	No blood-negative
1	1-5%	Changed formed pellets	Blood positive-trace reaction
2	5-10%	Loose/soft stool	Positive-moderate reaction
3	10-15%	Diarrhea	Positive-gross reaction
4	>15%		Gross rectal bleeding

*measured using Hemoplus Kit (Starstedt)

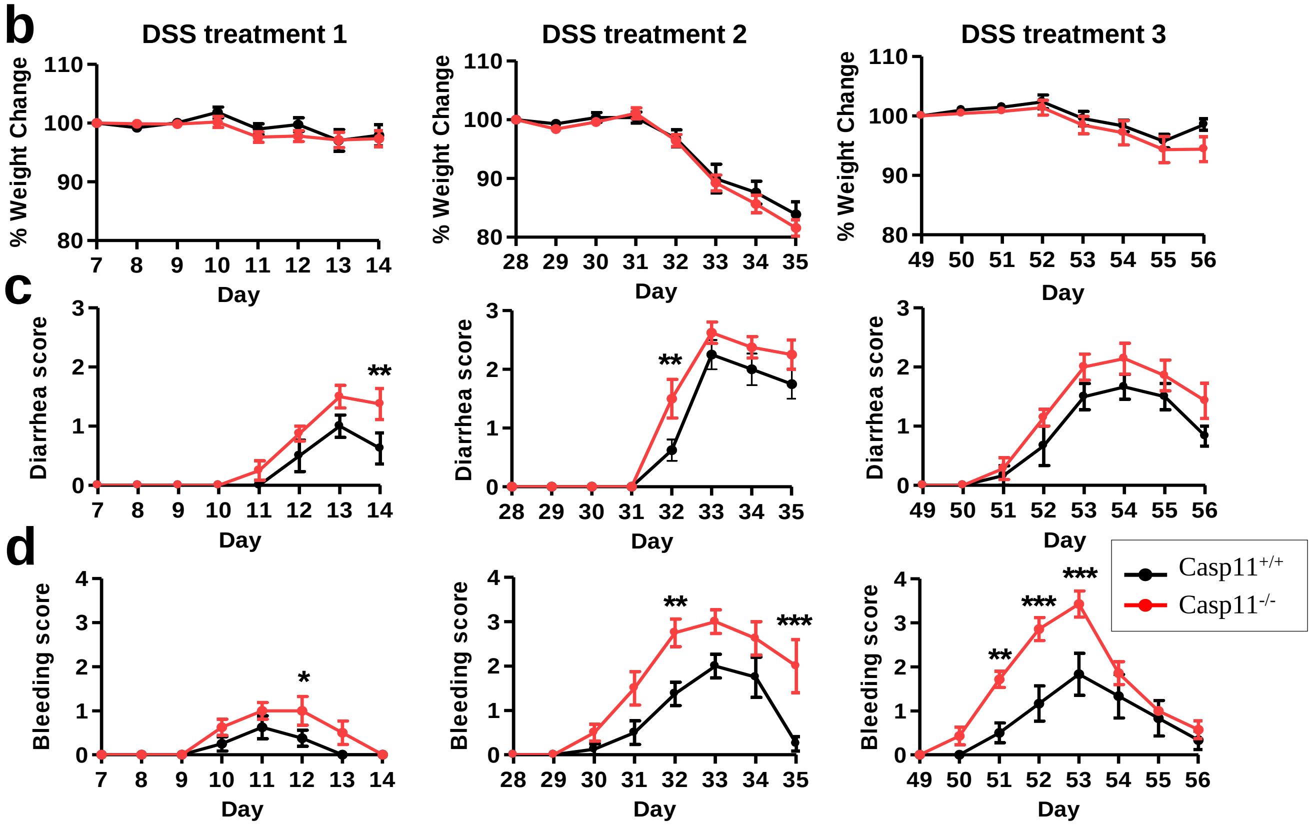

Supplementary Figure 1. Increased susceptibility of *Casp11*$^{-/-}$ mice to symptoms following AOM-DSS administration. (a) Table showing the scoring system used to measure disease-related symptoms of weight loss, diarrhea, and intestinal bleeding during AOM-DSS administration. Scores obtained for **(b)** weight loss; **(c)** diarrhea; and **(d)** fecal blood in a representative 15 week AOM-DSS trial. These scores were used to generate the disease activity index (DAI) score during/after DSS administration (corresponding to DAI scores in **Figure 1b**). Data represents mean ± SEM of n=6 AOM/DSS treated mice for both groups; *$p < 0.05$; **$p < 0.01$; ***$p < 0.001$ (two-way ANOVA followed by the Bonferroni post test).
