# Supplementary material for: Caspase-11 regulates the tumour suppressor function of STAT1 in a murine model of colitis-associated carcinogenesis
Source: Oncogene. 2018 Dec 11;38(14):2658–74. doi: 10.1038/s41388-018-0613-5 (PMC6484510; doi:10.1038/s41388-018-0613-5)
Supplement: Supplementary file 2 — Supplementary Figure 2 [file 41388_2018_613_MOESM2_ESM.pdf]

a

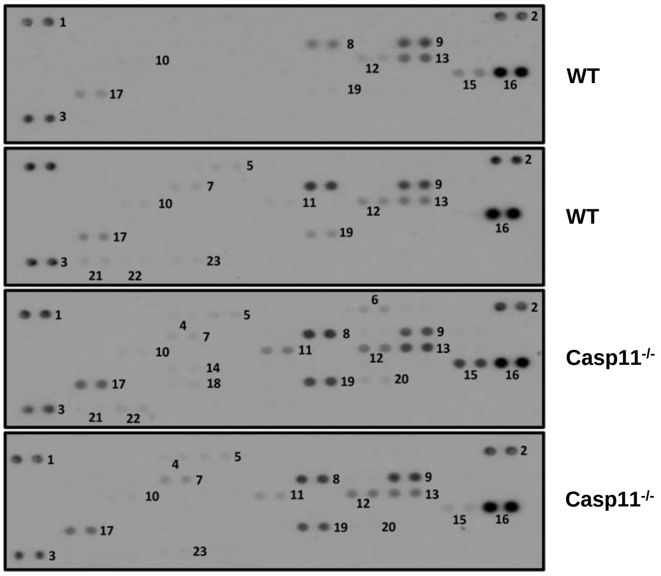

b

Array co-ordinate	Target protein	Change detected in Casp-11$^{-/-}$ mice
**1, 2, 3**	Reference spots	
**4**	Amphiregulin	+/+
**5**	Angiogenin	+/+
**6**	Coagulation Factor III	+/+
**7**	CD26	+/+
**8**	Endostatin	+/+
**9**	FGF Acidic	-/+
**10**	CX3CL1	+/+
**11**	HGF	+/+
**12**	IGFBP-2	+/+
**13**	IGFBP-3	+/+
**14**	KC	+
**15**	MMP9	+/-
**16**	NOV	+/-
**17**	Osteopontin	+/+
**18**	PDGF-AA	+/-
**19**	Platelet Factor IV	+/+
**20**	PIGF-2	+/+
**21**	SDF-1	-/-
**22**	Serpin E1	+/-
**23**	Serpin F1	-/-

Supplementary Figure 2. Enhanced angiogenesis-associated protein expression in Casp11$^{-/-}$, compared to WT, colons during CAC. (**a**) Proteome array of angiogenesis-associated proteins in duplicate WT and Casp-11$^{-/-}$ colon homogenates (300μg protein/blot) taken from mice on the final CAC trial day (d105). (**b**) Specific proteins captured on the array are labelled and identified, and proteins from Casp-11$^{-/-}$ colon homogenates which expression was increased (+) or decreased (-), compared to the protein's relative expression in WT colon homogenates, are annotated.
