# Supplementary material for: Caspase-11 regulates the tumour suppressor function of STAT1 in a murine model of colitis-associated carcinogenesis
Source: Oncogene. 2018 Dec 11;38(14):2658–74. doi: 10.1038/s41388-018-0613-5 (PMC6484510; doi:10.1038/s41388-018-0613-5)
Supplement: Supplementary file 3 — Supplementary Figure 3 [file 41388_2018_613_MOESM3_ESM.pdf]

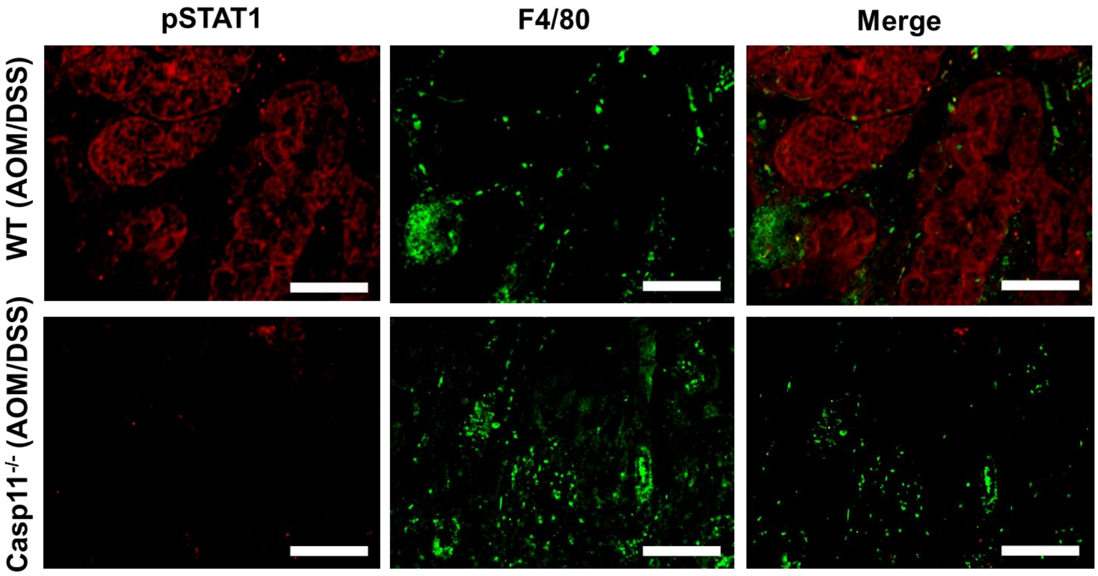

Supplementary Figure 3: STAT1 activation does not occur in F4/80 positive macrophages in tumorigenic colon tissue. Representative immunofluorescent images of pSTAT1 and macrophage marker F4/80 (Biorad) in distal colon sections from AOM/DSS treated WT and Casp11-/- mice at day 105. Scale bar = 20 µm.
