# Supplementary material for: Caspase-11 regulates the tumour suppressor function of STAT1 in a murine model of colitis-associated carcinogenesis
Source: Oncogene. 2018 Dec 11;38(14):2658–74. doi: 10.1038/s41388-018-0613-5 (PMC6484510; doi:10.1038/s41388-018-0613-5)
Supplement: Supplementary file 4 — Supplementary Figure 4 [file 41388_2018_613_MOESM4_ESM.pdf]

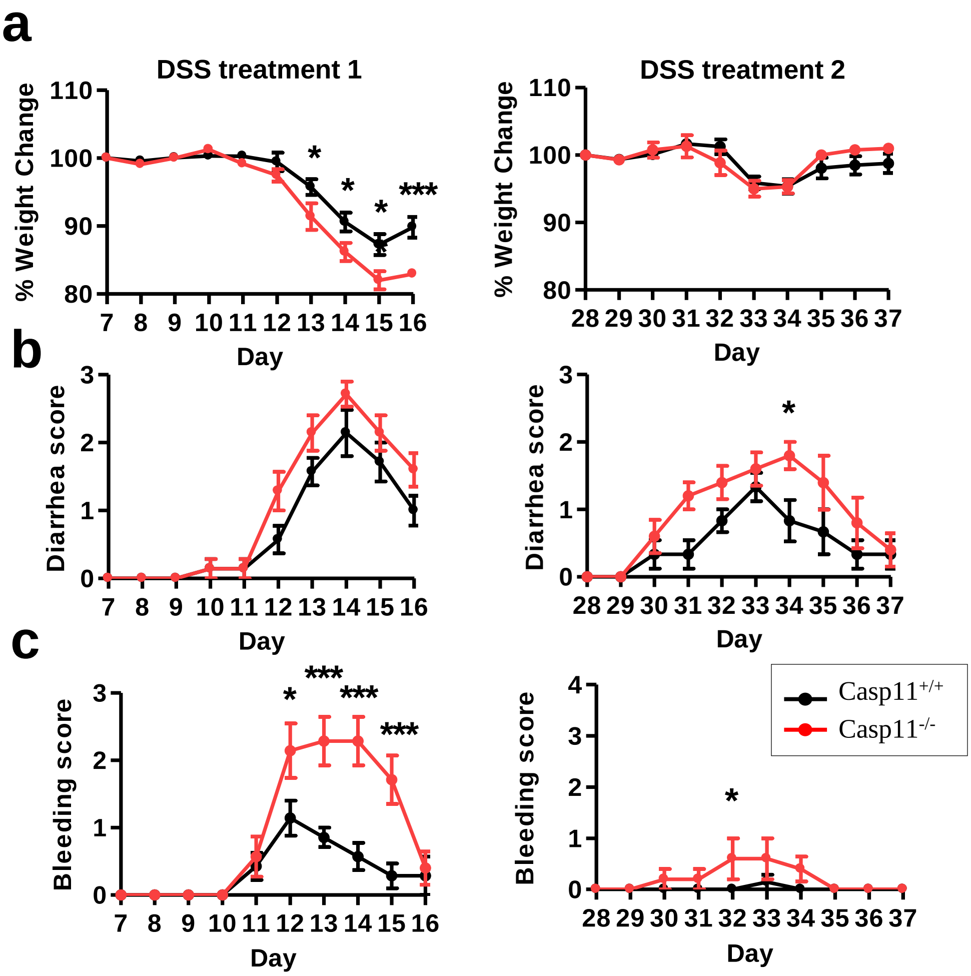

Supplementary Figure 4. Increased susceptibility of *Casp11*$^{-/-}$ mice to symptoms during 6 week AOM-DSS trial. Scores obtained for **(a)** weight loss; **(b)** diarrhea; and **(c)** fecal blood in a representative 6 week AOM-DSS trial. These scores were used to generate the disease activity index (DAI) score during/after DSS administration (corresponding to DAI scores in **Figure 5b**). Data represents mean ± SEM of n=6 AOM/DSS treated mice for both groups; $*p < 0.05$; $**p < 0.01$; $***p < 0.001$ (two-way ANOVA followed by the Bonferroni post test).
