# Supplementary material for: Caspase-11 regulates the tumour suppressor function of STAT1 in a murine model of colitis-associated carcinogenesis
Source: Oncogene. 2018 Dec 11;38(14):2658–74. doi: 10.1038/s41388-018-0613-5 (PMC6484510; doi:10.1038/s41388-018-0613-5)
Supplement: Supplementary file 5 — Supplementary Figure 5 [file 41388_2018_613_MOESM5_ESM.pdf]

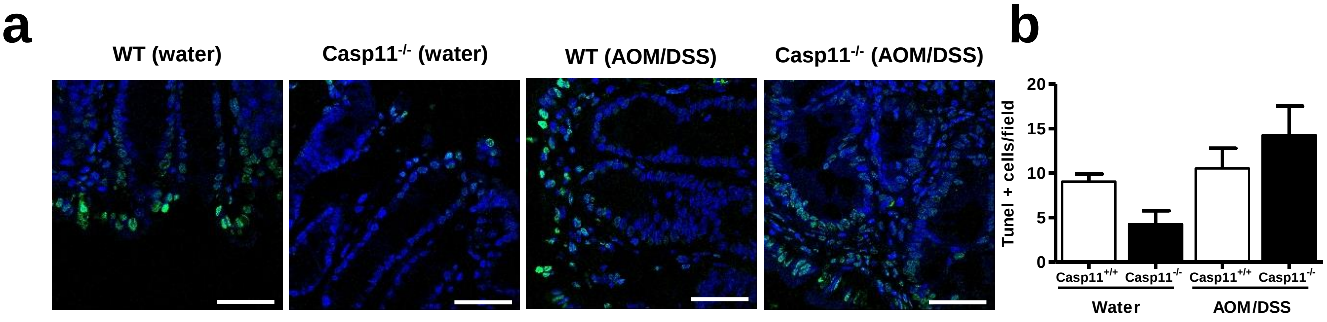

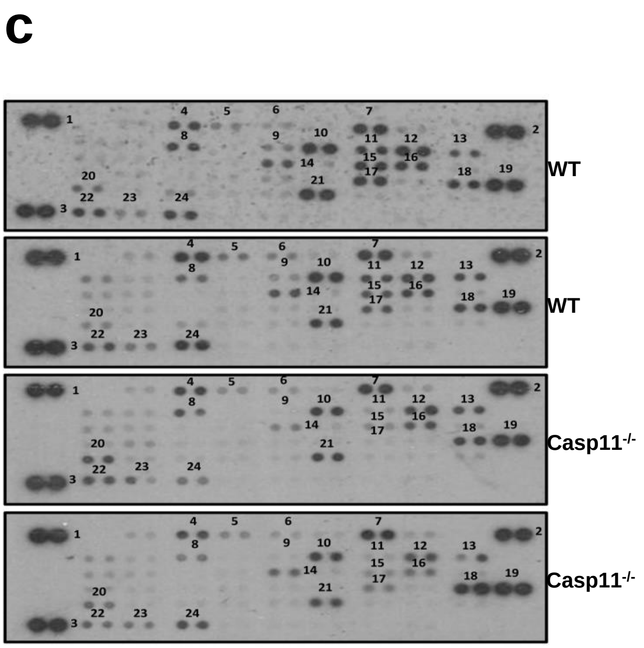

d

Array co-ordinate	Target protein	Detected change in Casp-11$^{-/-}$ mice
1, 2, 3	Reference spots	
4	Amphiregulin	-/-
5	Angiogenin	-/-
6	Angiopoietin-1	-/-
7	Coagulation Factor III	-/-
8	CD26	-/-
9	Endoglin	-/-
10	Endostatin	-/-
11	Endothelin-1	-/-
12	FGF Acidic	-/-
13	FGF Basic	-/-
14	HGF	-/-
15	IGFBP-2	-/-
16	IGFBP-3	-/-
17	MMP-3	-/-
18	MMP-9	+/+
19	NOV	-/+
20	Osteopontin	+/+
21	Platelet Factor IV	-/-
22	SDF-1	-/-
23	Serpin E1	-/-
24	Serpin F1	-/-

Supplementary Figure 5. Cell death and angiogenesis-associated protein expression during CAC development. (**a**) Representative fluorescent staining images of TUNEL^{+} cells from distal colon tissue sections of AOM/DSS treated WT and Casp11$^{-/-}$ mice sacrificed on day 42 of the CAC trial. Data represents mean ± SEM of n=5 AOM/DSS treated mice for both groups (two tailed independent student *t*-test). (**b**) Quantification of TUNEL^{+} cells shown in (**a**). (**c**) Proteome array analysis of angiogenesis-associated proteins in colon homogenates of AOM/DSS treated WT and Casp-11$^{-/-}$ mice (300 μg protein/blot) taken from mice at day 42 of the CAC trial. (**d**) Specific proteins captured on the labelled array are identified, and proteins from Casp-11$^{-/-}$ colon homogenates which expression was increased (+) or decreased (-), compared to the protein's relative expression in WT colon homogenates, are annotated.
