# Supplementary material for: Caspase-11 regulates the tumour suppressor function of STAT1 in a murine model of colitis-associated carcinogenesis
Source: Oncogene. 2018 Dec 11;38(14):2658–74. doi: 10.1038/s41388-018-0613-5 (PMC6484510; doi:10.1038/s41388-018-0613-5)
Supplement: Supplementary file 6 — Supplementary Figure 6 [file 41388_2018_613_MOESM6_ESM.pdf]

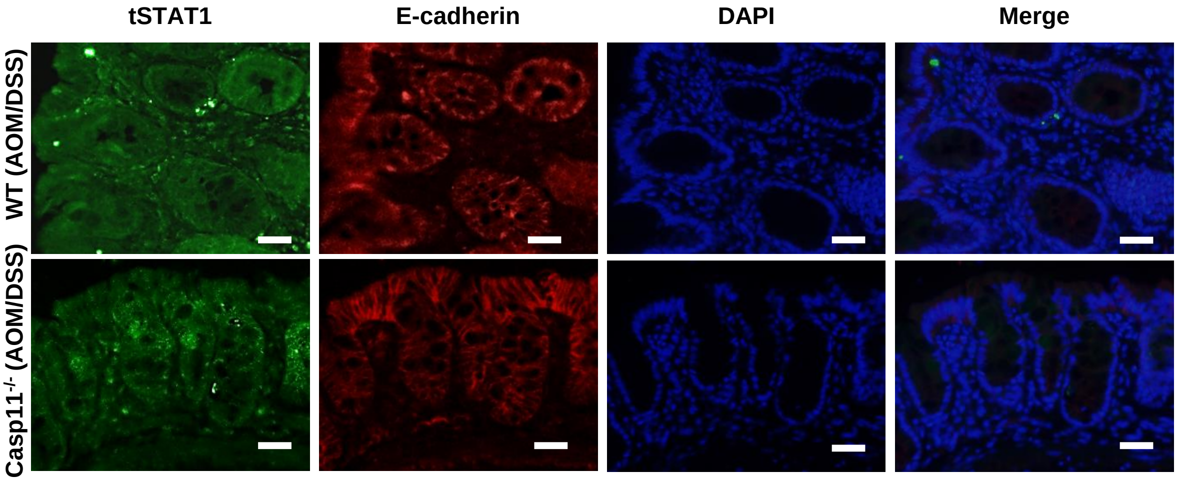


**Supplementary Figure 6: IF staining for total STAT1 in dysplastic colon tissue from WT and *Casp11*$^{-/-}$ mice.** Representative IF images of distal colon sections from WT/Casp11$^{-/-}$ mice at day 42 post-AOM/DSS. Staining for total STAT1 is shown, with co-staining for epithelial marker E-cadherin and DAPI (Scale bar = 20 μm).
